# Note on Erythrophlæin—Another Supposed Local Anæthetic

**Published:** 1888-05

**Authors:** Alvin A. Hubbell

**Affiliations:** Professor of Diseases of the Eye, Ear and Throat, Medical Department of Niagara University, Buffalo, N. Y.


### *NOTE ON ERYTHROPHLÆIN—ANOTHER SUPPOSED LOCAL ANÆSTHETIC.*

By ALVIN A. HUBBELL, M. D.,

Professor of Diseases of the Eye, Ear and Throat, Medical Department of Niagara University, Buffalo, N. Y.

On January 11, 1888, Lewin communicated to the Berlin Medical Society his observations on local anæsthesia, induced by the application to the surface of mucous membranes and by subcutaneous injection of solution of erythrophlæin hydrochlorate, an alkaloid extracted from the bark of an African tree—erythrophlæum judiciale (erythrophlæum guineense).

This drug, he stated, when applied to the eye in solutions of the strength of one-twentieth to one-fourth of one per cent., produced anæsthesia of the cornea and conjunctiva "in from fifteen to twenty minutes, which lasts from one to two and a-half days." More concentrated solutions "cause a typical and very intense irritation, followed by dense opacities of the cornea." Used hypodermically, it produced profound anæsthesia in the parts which it reached.

Since this announcement was made, numerous observers have examined the properties of the drug. Some seem to corroborate Lewin's observations, while others do not.

Immediately after learning of Lewin's statements, I obtained a specimen of the erythrophlæin, and, in solutions of one-twentieth to one-tenth per cent., have experimented with it both on the eyes of animals and men. The results have been, uniformly, a peculiar and intense smarting pain, which continues for twenty to thirty minutes after instillation, causing conjuncl injection, lachrymation, and hyperæsthesia of the parts.

I have not been able to detect any anæsthesia at any stage of its action. The pupil remains unchanged, and the vision does not appear to be affected in any way.

The failure to obtain anæsthesia may be due to the drug which I have used not being genuine or of good quality; but I procured it from a reliable source, as I believe, and is said to be the same as that used by others who claim to get the anæsthetic effects. Future observations, and with other specimens of the drug, may yield different results. But erythrophlæin has not yet proved itself to me to be a local anæsthetic.

